# Supplementary material for: Pattern formation in reaction–diffusion system on membrane with mechanochemical feedback
Source: Sci Rep. 2020 Nov 11;10:19582. doi: 10.1038/s41598-020-76695-x (PMC7659017; doi:10.1038/s41598-020-76695-x)
Supplement: Supplementary file 1 — Supplementary Information 1. [file 41598_2020_76695_MOESM1_ESM.pdf]

Supplementary Material: Pattern Formation in Reaction-Diffusion System on Membrane with Mechanochemical Feedback

*Naoki Tamemoto and Hiroshi Noguchi**

Institute for Solid State Physics, University of Tokyo, Kashiwa, Chiba 277-8581, Japan

CALCULATION OF DOMAIN SIZE AND LOCALLY AVERAGED CURVATURE

A Turing pattern is considered to be formed when the minimum and maximum values of u are more than 0.2 apart. We designate one region as the domain for the calculation of the number of domains N_{d} and the size of one domain S_{d} if u is greater than the mean value of the maximum and minimum values and is connected by the bond network.

When evaluating the local curvature, we calculated $\widetilde{H}$ by averaging the curvature H of the adjacent nodes up to depth 2 to reduce temporal thermal fluctuations. Without smoothing (i.e., smoothing depth = 0), the probability distribution of the local curvature is broad, and thus the dependence of the maximum values of local curvature $\widetilde{H}_{\mathrm{max}}$ on the diffusion constants D_u is unclear (Figs. S1(b) and (c)). According to the probability distributions and the time evolutions of $\widetilde{H}_{\mathrm{max}}$, the shape differences can be distinguished well at the smoothing depth 2 or above (Figs. S1(b) and (c)). Thus, we chose the smoothing depth 2 because the local curvature can be evaluated and the summed area for smoothing is small (Fig. S1(a)).

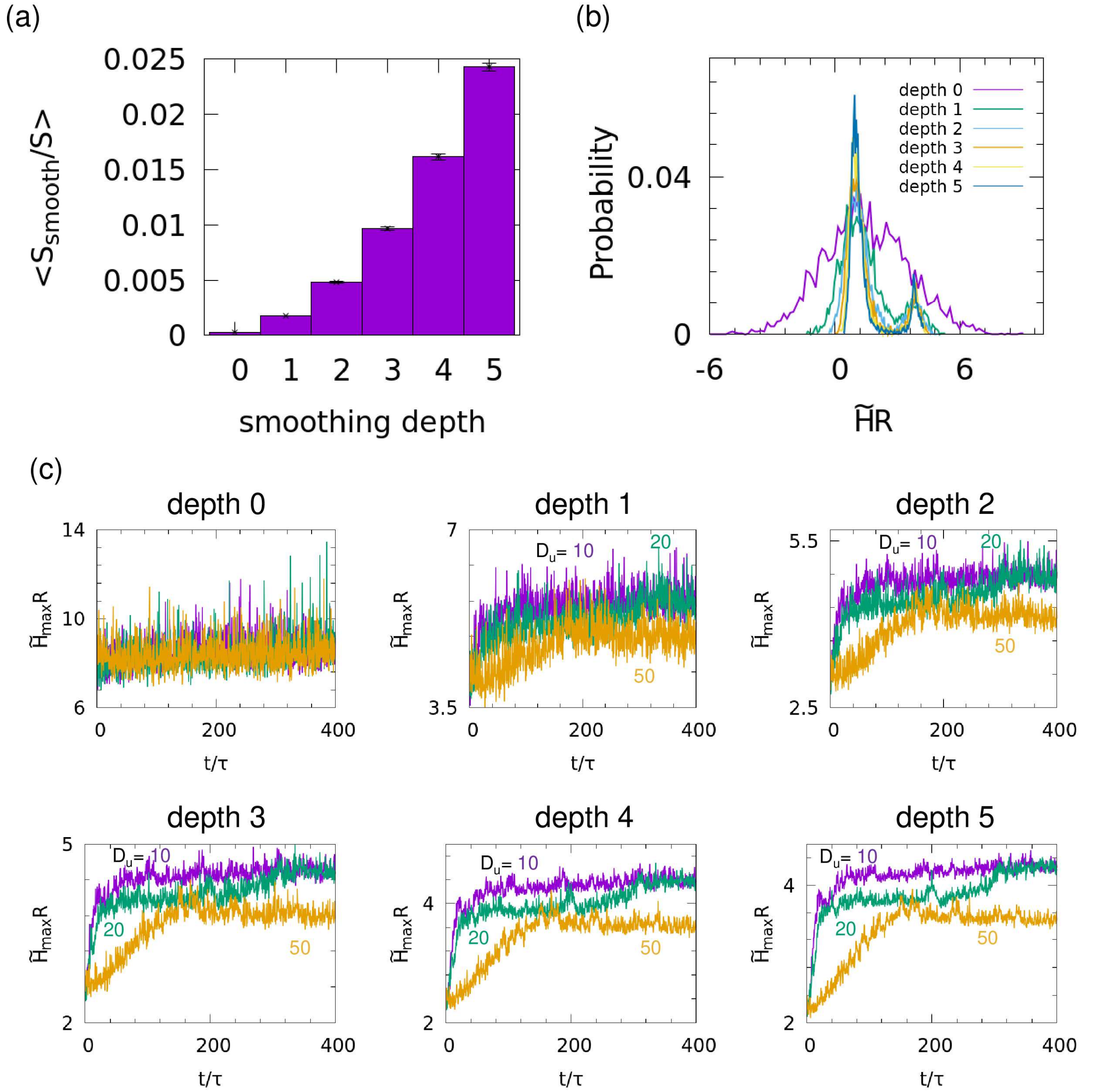

Figure S1. Dependence on smoothing depth to calculate the local curvature $\widetilde{H}$ for the data shown in Fig. 5(h). (a) The average of a summed-area smoothed at various smoothing depths, S_{smooth} as a proportion of total area S. (b) Probability distributions of the local curvature at various smoothing depths. The condition is the same as in (a). (c) Time evolutions of the maximum values of local curvature at various smoothing depths.

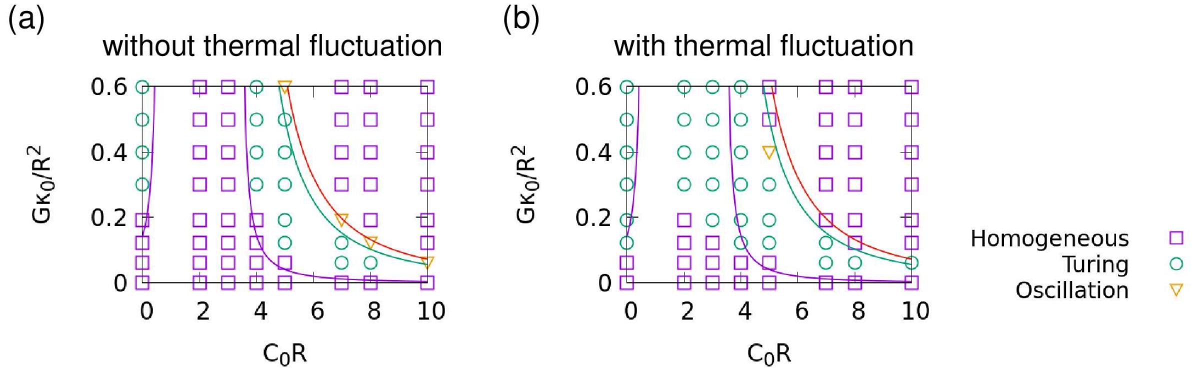

Figure S2. (a, b) Phase diagrams for $A = 4.5$, $B = 2.02$, $\eta = 0.1$, $D_u = 20$, and $V^* = 1$ without thermal fluctuations (a) and with thermal fluctuations (b). The purple and green lines on the phase diagrams represent the Turing bifurcation curve and the Hopf bifurcation, respectively. The symbols represent the simulation results. The red line indicates $A + A' = 0$.

THE EFFECT OF THERMAL FLUCTUATIONS

We performed simulations with and without thermal fluctuations at $A = 4.5$, $B = 2.02$, $\eta = 0.1$, $D_u = 20$, and $V^* = 1$ to investigate the effect of temporal thermal fluctuations (Fig. S2). Since the area and volume of the vesicles are constrained by the harmonic potentials, the reduced volume fluctuates only slightly, with a standard deviation of $\Delta V^* = 0.00004$. The results of the simulation without thermal fluctuations are consistent with those of the linear stability analysis. In contrast, for simulations conducted with thermal fluctuations, the results showing large G are different from those of both the linear stability analysis and the simulation without thermal fluctuations (Figs. S2(a) and (b)). Thus, the stable phase is modified even by small membrane fluctuations.

This fluctuation effect is caused by the dependence of A' on H. Since $A' = -G\left((\kappa_1 - \kappa_0)(2H)^2/2 - 2\kappa_1 C_0 H + \kappa_1 {C_0}^2/2\right)$, the average value of $A + A'$ in the simulation with thermal fluctuations is reduced by the variance of local curvature H. For example, in the simulation for $G\kappa_0/R^2 = 0.60$ and $C_0R = 2$, the time-averaged values ± standard error of $A + A'$ are 3.83 ± 0.01 and 5.69 in the presence and absence of thermal fluctuations, respectively. The latter value is in agreement with the theoretical value of 5.69. The shift caused by the thermal fluctuations can be understood by the variance of the local curvature. When we eliminate the variance effect, the difference is removed as $A + A' + G(\kappa_1 - \kappa_0)\Delta(2H)^2/2 = 5.69 \pm 0.0004$, where $\Delta(2H)^2$ is the variance of the local curvature. Since the critical value of $A + A'$ for Turing bifurcation is 4.21, the Turing patterns appear in the presence of thermal fluctuations. This effect becomes larger as G

increases, and thus the phase diagram with thermal fluctuations shows larger deviations at a larger value of G.

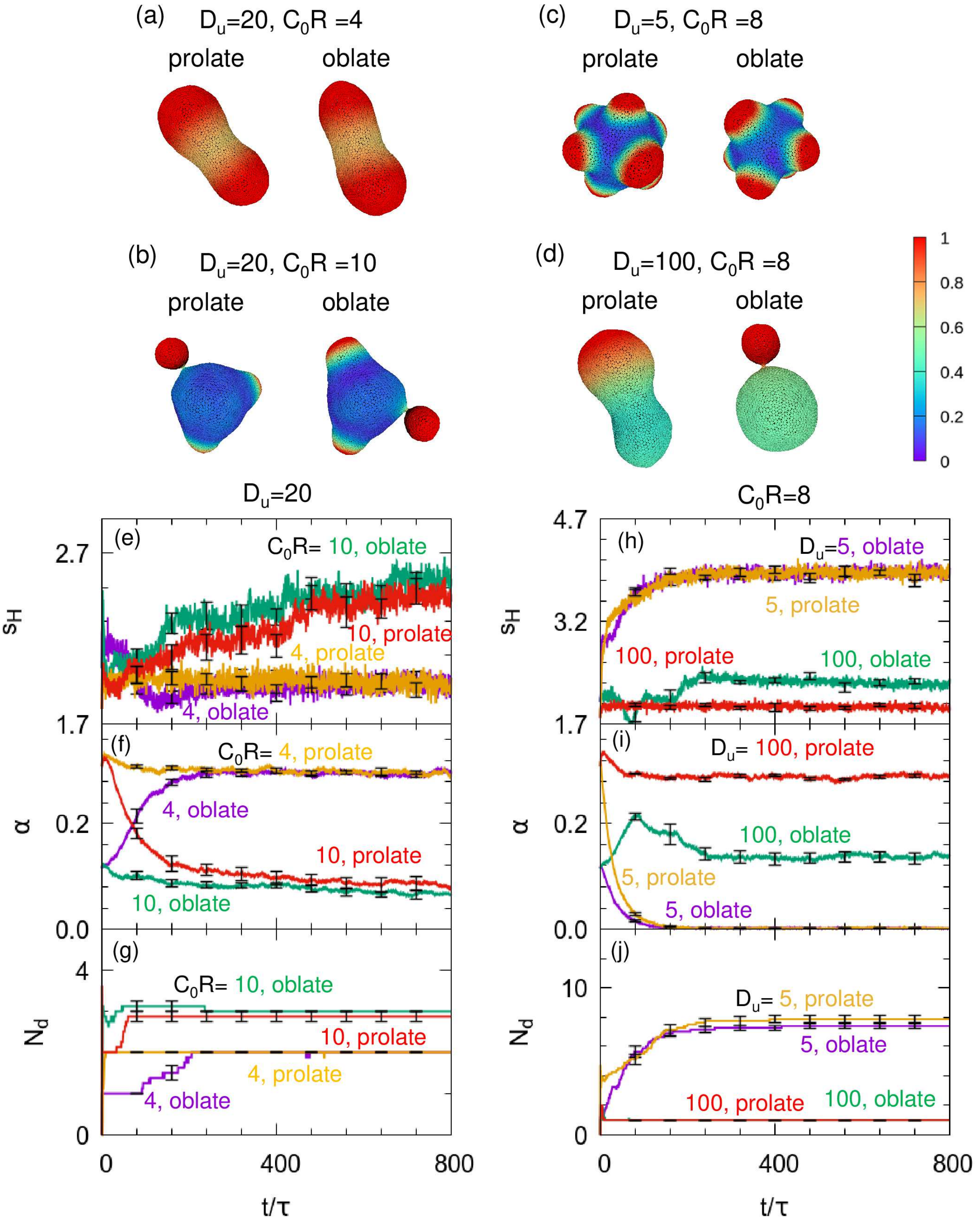

Figure S3. (a–d) Snapshots of vesicles for $A = 4.5$, $B = 2.02$, $\eta = 0.1$, $G\kappa_0/R^2 = 0.046$, and $V^* = 0.8$ for two more values of D_u and C_0 starting from prolate and oblate shapes. (a) $D_u = 20$ and $C_0R = 4$. (b) $D_u = 20$ and $C_0R = 10$. (c) $D_u = 5$ and $C_0R = 8$. (d) $D_u = 100$ and $C_0R = 8$. The color indicates the concentration of curvature-inducing protein, u. (e–

j) Time evolution of (e, h) the separation metric of the local curvature, s_H, (f and i) asphericity, α, and (g, j) the number of domains, N_d. The data for $C_0R = 4$ and 10 at $D_u = 20$ are shown in (e–g), and the data for $D_u = 5$ and 100 at $C_0R = 8$ are shown in (h–j). The orange and red lines indicate simulations starting from prolate shapes, and the purple and green lines indicate simulations starting from oblate shapes. Results are presented as mean ± standard error (n = 7 for $D_u = 100$ and $C_0R = 8$ started from oblate form. Otherwise n = 8).

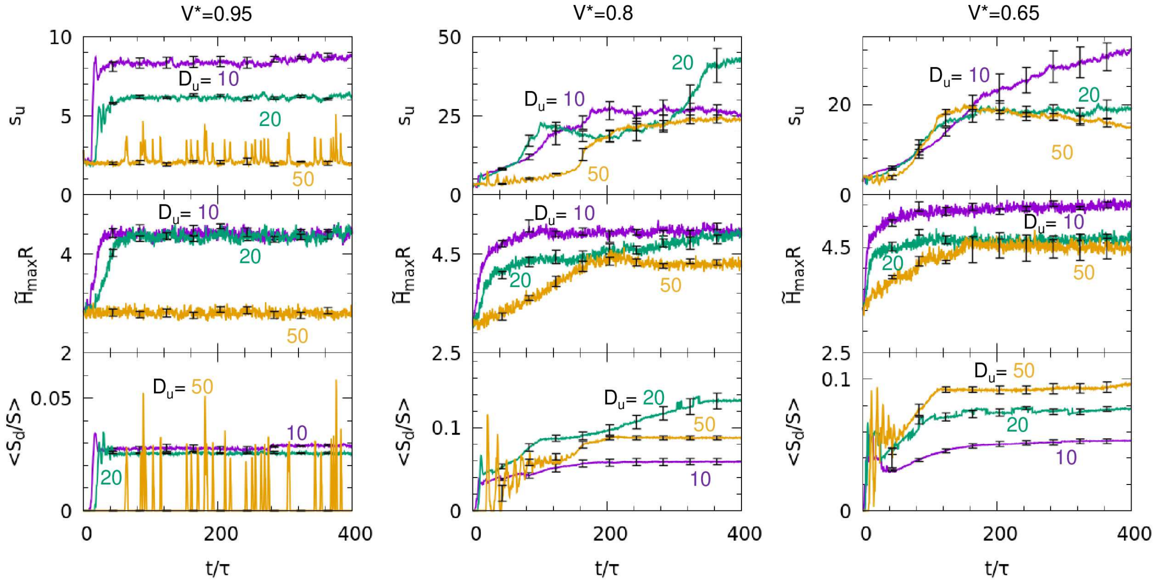

Figure S4. Average time evolution of the separation metrics, s_u, the maximum value of the local curvature, $\widetilde{H}$, and the domain area ratio $\langle S_\mathrm{d}/S\rangle$. Results are presented as the mean ± standard error (n = 8). The parameters are the same as in Fig. 5.

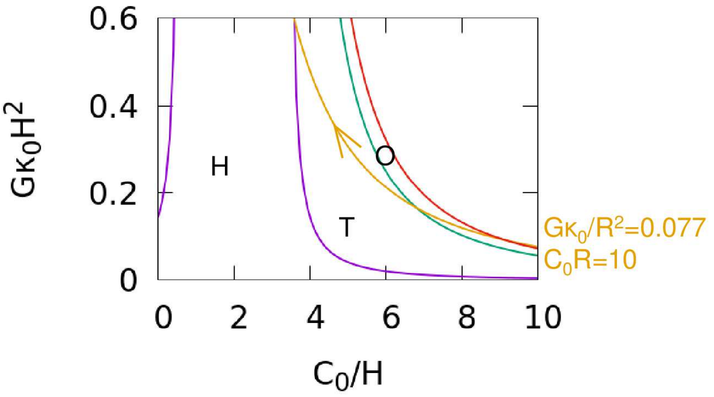

Figure S5. The phase diagram for the Brusselator, modified to include a membrane curvature effect, for $A = 4.5$, $B = 2.02$, and $\eta = 0.1$. The purple, green, and red lines are the same as those shown in Fig. 1. The orange line indicates stability in changing local curvature H at $G\kappa_0/R^2 = 0.077$ and $C_0R = 10$. As H increases, it is shifted toward the upper left, and the transition from oscillation to Turing mode occurs.

MOVIE CAPTIONS:

Movie S1. Shape transformation of a vesicle from a prolate shape to a three-spindle shape according to the Turing pattern. Each frame step is 200τ. The data are the same as in Fig. 3(a).

Movie S2. Transition from the temporal oscillating pattern to the stable Turing pattern. Each frame step is 40τ. The data are the same as in Fig. 5(b), $D_u = 50$.

Movie S3. The formation of a stable small spot pattern. Each frame step is 10τ. The data are the same as in Fig. 6(a).

Movie S4. The formation of a transient large spot pattern. Each frame step is 10τ. The data are the same as in Fig. 6(b).
